# Cundurango a Failure

**Published:** 1871-12

**Authors:** Frank A. Stanford

**Affiliations:** Columbus, Georgia


### CUNDURANGO A FAILURE.

We publish the following additional testimony in regard to the utter worthlessness of cundurango, from one of the most distinguished and reliable surgeons and physicians in Georgia. That its introduction to the profession was a deliberate swindle upon the part of a set of cruel impostors, the evidence is overwhelming:

Columbus, Georgia, December 17th, 1871.

*Editors Atlanta Medical and Surgical Journal:*

Without troubling you to publish at great length the particulars of two cases—one of scirrhus breast, which I removed some months ago, and in which the disease returned; the other a case of epithelioma, so extensive as to involve the tissues generally of the left mammary region, and in which I declined to operate—I will simply state, that after a long and expensive use of the cundurango, it has utterly failed to accomplish the least good. The article used was obtained from Bliss, Keen & Co., New York, by one of our druggists, Mr. J. J. Griffin. Very respectfully, your obedient servant,

Frank A. Stanford, M.D.

---

Chloral in Cod-Liver Oil (Medical Times) is said to render it much less nauseous, and prevents the night-sweats of the phthisical patient, induces sleep, and creates apetite. The pure chloral hydrate crystals may be added to cod-liver oil in the proportion of ten grains of the former to one hundred and ninety of the latter.

